# Climatic records and within field data on yield and harvest quality over a whole vineyard estate

**DOI:** 10.1016/j.dib.2023.109579

**Published:** 2023-09-16

**Authors:** Jean-Philippe Gras, Guilhem Brunel, Arnaud Ducanchez, Thomas Crestey, Bruno Tisseyre

**Affiliations:** ITAP, Univ. Montpellier, INRAE, Institut Agro, 2 Place Pierre Viala 34060 Montpellier, France

**Keywords:** Precision viticulture, Grapes quality, Yield calculation, Meteorological data, GNSS

## Abstract

Detailed and precise knowledge of production parameters (yield, quality, health status, etc.) in agriculture is the basis for analyzing the effect of any agricultural practice. Fine mapping of production parameters makes it possible to identify the origin of observed variability, whether associated with environmental factors or with agricultural practices. In viticulture, in real commercial context, these data are rare because monitoring systems embedded on harvesting machines for grape yield and quality are not yet available. As a result, they are costly and/or cumbersome to acquire manually. As an alternative, a research project has been proposed to test low-cost methods using GNSS tracking devices for yield and harvest quality mapping in viticulture. The data set was acquired as part of this research. The methodology was applied on a commercial vineyard of 30 ha during the whole 2022 harvest season. The method has identified harvest sectors (HS) associated to measured production parameters (grape mass and harvest quality parameters: sugar content, total acidity, pH, yeast assimilable nitrogen, organic nitrogen) and calculated production parameters (potential alcohol of grapes, yield, yield per plant, percentage of unproductive plants) over the entire vineyard. The grape mass was measured at the vineyard cellar or at the wine-growing cooperative by calibrated scales. The harvest quality parameters were measured from samples on grape must at a commercial laboratory specialized in oenological analysis (Institut Coopératif du Vin, Montpellier, France) with standardized protocols. The percentage of unproductive plants of a harvest sector was calculated from the manually geolocation of each unproductive plants (dead plants + missing plants) over the entire vineyard, the plantation density of blocks, and the geolocalization of the harvest sector. The mean area of these harvest sectors is 0.3 ha. The data set is supplemented by climatic data from a weather station deployed in the center of the vineyard. It provided three climatic parameters (relative humidity, rainfall, air temperature) every 15 min, for the 2020, 2021 and 2022 years. It was also supplemented by a complete description of the vineyard blocks (grape variety, plantation year, area, inter-row distance and vine distance). The proposed data set constitutes a unique and interesting resource for research in agronomy, vine ecophysiology and remote sensing. It can be used for any research in vine ecophysiology aimed at identifying potential relationships between yield and harvest quality parameters for different grape varieties. The data set only covers one year, which is a limitation for studying inter-annual variability of the parameters measured. Another limitation of the method concerns the footprint (0.3 ha on average) of the parameters measured.

Specifications Table

**Table:** 

Subject	Agronomy and Crop Science

Specific subject area	Precision viticulture
Type of data	Spatial data files (consisting of .shp and associated files) Text table (.csv)
How the data were acquired	Agronomical data were acquired during the 2022 harvest season. Grape mass was obtained from harvest trailers weighing measurements at the winery or at the wine-growing cooperative by calibrated scales. Sugar content, total acidity, pH, yeast assimilable nitrogen, organic nitrogen were obtained from grape must sample analyses, in a commercial laboratory specialized in oenological analysis (Institut Coopératif du Vin, Montpellier, France), with standardized protocols. The weather data was acquired with a weather station (Agriscope, Montpellier, France) installed in the center of the vineyard. Block data boundaries (they correspond to the regulatory contour used in France to declare the grapes planted areas) were measured with a real-time kinematic (RTK) GNSS receiver [[1], [2]]. Inter-row distance and vine-distance were measured with a measuring tape at the plantation time. Unproductive vines were geolocalized manually over the whole vineyard using a RTK GNSS receiver, of accuracy 1-5 cm.
Data format	Raw and Analyzed
Description of data collection	The dataset was obtained over a whole commercial wine estate located in the south of France. For Agronomic data, the measured data (mass of grape, harvest quality parameters) were spatially reallocated to intra-blocks geometry using the geolocation of harvest machines (Algorithm in Python). The weather data were obtained during the 2020, 2021, and 2022 years every 15 minutes using the weather station of the company Agriscope (Montpellier, France). Block data boundaries and attributes (year of plantation, variety, area, inter-row distance and vine distance), as well as the geolocation of unproductive wines were obtained from the vineyard Farm Management Information System.
Data source location	Institution: Institut Agro Montpellier City: Montpellier Country: France
Data accessibility	Repository name: Zenodo Data identification number:10.5281/zenodo.8328384 Direct URL to data: https://doi.org/10.5281/zenodo.8328384
Related research article	J-P. Gras, S. Moinard, T. Crestey and B. Tisseyre. Mapping grape yield with low-cost vehicle tracking devices, In Precision agriculture’23*,* Wageningen Academic Publishers. (2023) 555-561. https://doi.org/10.3920/978-90-8686-947-3_70

## 1 Value of the Data

The dataset presented in this paper is a particularly exhaustive dataset with a spatial description of grape production parameters (yield, yield per plant and six harvest quality parameters) over a whole vineyard. This dataset associates georeferenced harvest sectors (HS) of mean size 0.3 ha to grape production parameters. The data set is supplemented by climatic data from a weather station deployed on the vineyard which provides three main climatic parameters every 15 min, over the 2020, 2021 and 2022 years. It was also supplemented by a complete description of the vineyard blocks (grape variety, plantation year, area, inter-row distance and vine distance).

• The dataset can be used for any research in vine ecophysiology aimed at identifying potential relationships between yield and harvest quality parameters for different grape varieties [3].
• It can also be used to explore, test and validate modeling approaches that aim at understanding or predicting yield and/or grape quality parameters according to the year's climatic data [4].
• Another value of this data set is the spatial footprint of the georeferenced data which makes it possible to relate multispectral time series of satellite images such as Sentinel to identify potential links between yield and harvest quality parameters and remote sensed canopy changes [5].

## 2 Data Description

The data were obtained on a 30 ha vineyard located in the south of France (Villeneuve-lès-Maguelone, France; 43.532300° N, 3.864230° E). The data includes 3 types of data:

• Block(s) data: the data describes each vineyard block. The description parameters of the block are the identity, the year of plantation, the variety of the grapes, the area, the inter-row distance and vine-distance. The data is provided as spatial data files (.shp and associated files) using the WGS84 global coordinate reference system for latitude and longitude. It is composed of 68 features. Each feature describes a block geometry and associated attributes, listed and explained in Table 1. Some blocks of this vineyard are used for research purposes (experiment on grapes varieties). This explains that some blocks have a very small area (between 0.02 to 0.2 ha).

**Table 1:** Description of vineyard blocks variables. Each line corresponds to a vineyard block.

Variable name	Description	Unit
BlockId	**Block Identity**: an unique identity for each block	
Year	**Year:** year of grape plantation	date
Variety	**Variety:** variety of the grape	
Area	**Area**: block area	ha
IRow	**Inter-Row:** distance between two rows	m
VDistance	**Vine Distance**: distance between two plants in a same row	m
• Agronomic data: The data describes production parameters for each “harvest sector” for the 2022 harvest season. A harvest sector (HS) is the area covered by the harvester to fill a harvest trailer before its departure to a cellar. Measurement are done to characterize the grape crop on the trailer (see section “Experimental design, materials and methods” for more explanation). The harvest sector is therefore the spatial element inside blocks associated to the measured parameters. The mean area of these harvest sectors over the vineyard is equal to 0.3 ha. The description parameters of the harvest sector are the block id that it belongs to, the harvest date, yield parameters (mass, yield, and yield per plant), grape quality parameters (sugar, alcohol, total acidity, pH, yeast assimilable nitrogen and organic nitrogen), and the percentage of unproductive plants. All the parameters used to describe these harvest sectors are listed in Table 2. The data is provided as spatial data file (.shp and associated files) using the WGS84 global coordinate reference system for latitude and longitude. Each feature describes a harvest sector geometry and associated parameters. It is composed of 87 features each corresponding to the 87 harvest sectors. Harvest quality parameters (sugar, alcohol, total acidity, pH, yeast assimilable nitrogen and organic nitrogen) are only available for 50 features (must analysis was only done for trailer which deliver grapes at the winery cellar: see section “Experimental design, materials and methods” for more explanation).

**Table 2:** Description of variables associated to each harvest sector. Each line corresponds to a harvest sector.

Variable name	Description	Unit
HDay	**Harvest Day**: date of the harvest	DD/MM/YYYY
BlocksId	**Blocks identity**: list of blocks id associated to a harvest sector.	
Area	**Area:** Area of an harvest sector	ha
Mass	**Mass**: mass of grapes associated to the harvest sector. The mass is measured at he winery cellar (by a dynamic system of weighing developed by the CAPTELS society) or at the wine-growing cooperative by a calibrated scale Accuracies of the scales are +- 50 kg.	kg
Sugar	**Sugar Content**: massic concentration of Glucose plus Fructose in the must. It is measured on grape must at a commercial laboratory specialized in oenological analysis (Institut Coopératif du Vin, Montpellier, France). The measuring technique is Fourier Transform Infrared (FTIR) spectroscopy. The uncertainty of the measurement is 15 %.	g.l^−1^
Alcohol	**Potential Alcohol**: The alcohol content of a wine that will result from the complete fermentation of a must. It is calculated from the measured sugar content: Potential Alcohol = Sugar Content/16.83	% v/v
Tacidity	**Total acidity**: The total acidity represents the sum of all titratable acids in a must sample (mainly: tartaric, malic and citric). In that case, it is converted on a current acid scale: g.l^−1^ of sulfuric acid (H_2_SO_4_). It is measured on grape must at a commercial laboratory specialized in oenological analysis (Institut Coopératif du Vin, Montpellier, France). The measuring technique is Fourier Transform Infrared (FTIR) spectroscopy. The uncertainty of the measurement is 6 %.	g H_2_SO_4_.l^−1^
pH	**pH:** pH is a scale used to specify the acidity or basicity of an aqueous solution. The pH of a wine must is a measure of the concentration of free hydrogen ions in solution. It is measured on grape must at a commercial laboratory specialized in oenological analysis (Institut Coopératif du Vin, Montpellier, France). The measuring technique is Fourier Transform Infrared (FTIR) spectroscopy. The uncertainty is equal to 10 %.	unitless
YAN	**Yeast Assimilable Nitrogen**: The assimilable nitrogen is the combination of free amino nitrogen, ammonia (NH_3_) and ammonium (NH_4_^+^) that is available for a yeast. It is measured on grape must at a commercial laboratory specialized in oenological analysis (Institut Coopératif du Vin, Montpellier, France).	mg.l^−1^
ON	**Organic Nitrogen**: Percentage of nitrogen organic form in the must. It is measured on grape must at a commercial laboratory specialized in oenological analysis (Institut Coopératif du Vin, Montpellier, France).	%
Yield	**Yield**: Mass of grape associated to the harvest sector divided by the area of the harvest sector	t.ha^−1^
UPlants	**Unproductive Plants**: percentage of unproductive plants (dead plants + missing plants) in a harvest sector. It is calculated from the manually geolocation of each unproductive plants (dead plants + missing plants) over the entire vineyard, the plantation density of blocks, and the geolocalization of the harvest sector.	%
YieldPP	**Yield Per Plant**: mass of grape associated to a harvest sector divided by the number of productive plants in the harvest sector.	kg.plant^−1^
• Weather data: the data consists of meteorological data for the 2020, 2021 and 2022 years recorded by a weather station located in the center of the vineyard. The acquisition time step is 15 minutes. The recorded parameters are the followings: date, hour, relative humidity, rainfall and air temperature. The data are provided in Comma Separated Values (CSV) format. It is composed of 97988 lines. Each line describes meteorological data recorded at a given time. The attributes of each data acquisition are listed and explained in Table 3.

**Table 3:** Description of the weather variables recorded every 15 min from 01/01/2020 to 31/12/2022.

Variable name	Description(*sensors*)	Unit
Date	Date of the measurement	DD/MM/YYYY
Hour	Time of the measurement	HH:MM
RH %	**Relative Humidity**: p_v_/p_vsat_x100 (*Hygrometer*)	%
RAINFALL	**Rainfall**: amount of precipitation that fell during 15 min. (*Rain gauge*)	mm
AIR TEMP	**Air temperature**: measure of temperature. (*Thermometer*)	°C

The dataset files are presented in Table 4. These datasets are supplemented by three others files listed also in Table 4: “Metadata.xlsx”, “Yield Map.html”, “Yield_vs_variety.py”. “Metadata.xlsx” is metadata file which contains all information on the data to ease their use. “Yield Map.html” is a dynamic map showing the harvest sectors and their associated productions parameters. On this map, every time the mouse pointer enters a harvest sector, the harvest sector is colored in blue and a tooltip appears showing all production parameters associated with the harvest sector. “Yield_vs_variety.py” is a python script which illustrates an example of the potential use of the agronomical data. This code allows to load the data and join the agronomical and blocks data. In that example, the two datasets have been joined to associate grape variety(ies) (from the blocks data), to each harvest sector (from the agronomical data). Then a comparison of the productivity (yield) of three different and popular grape varieties (in south of France) is made.

**Table 4:** The data set files available.

Content	Folder	Format	File name
Metadata	Metadata	Excel table	Metadata.xlsx
Blocks data	Blocks data	Vector file	Blocks_data.cpg Blocks_data.dbf Blocks_data.prj Blocks_data.shp Blocks_data.shx
Agronomic data	Agronomic data	Vector file	Agronomical_data.cpg Agronomical_data.dbf Agronomical_data.prj Agronomical_data.shp Agronomical_data.shx
Weather data	Weather data	Text table	Weather data.csv
Yield map	Yield Map	Html file	Yield Map.html
Script	Script	Python script	Yield_vs_variety.py

## 3 Experimental Design, Materials and Methods

### 3.1 Experimental site

**Fig. 1:**
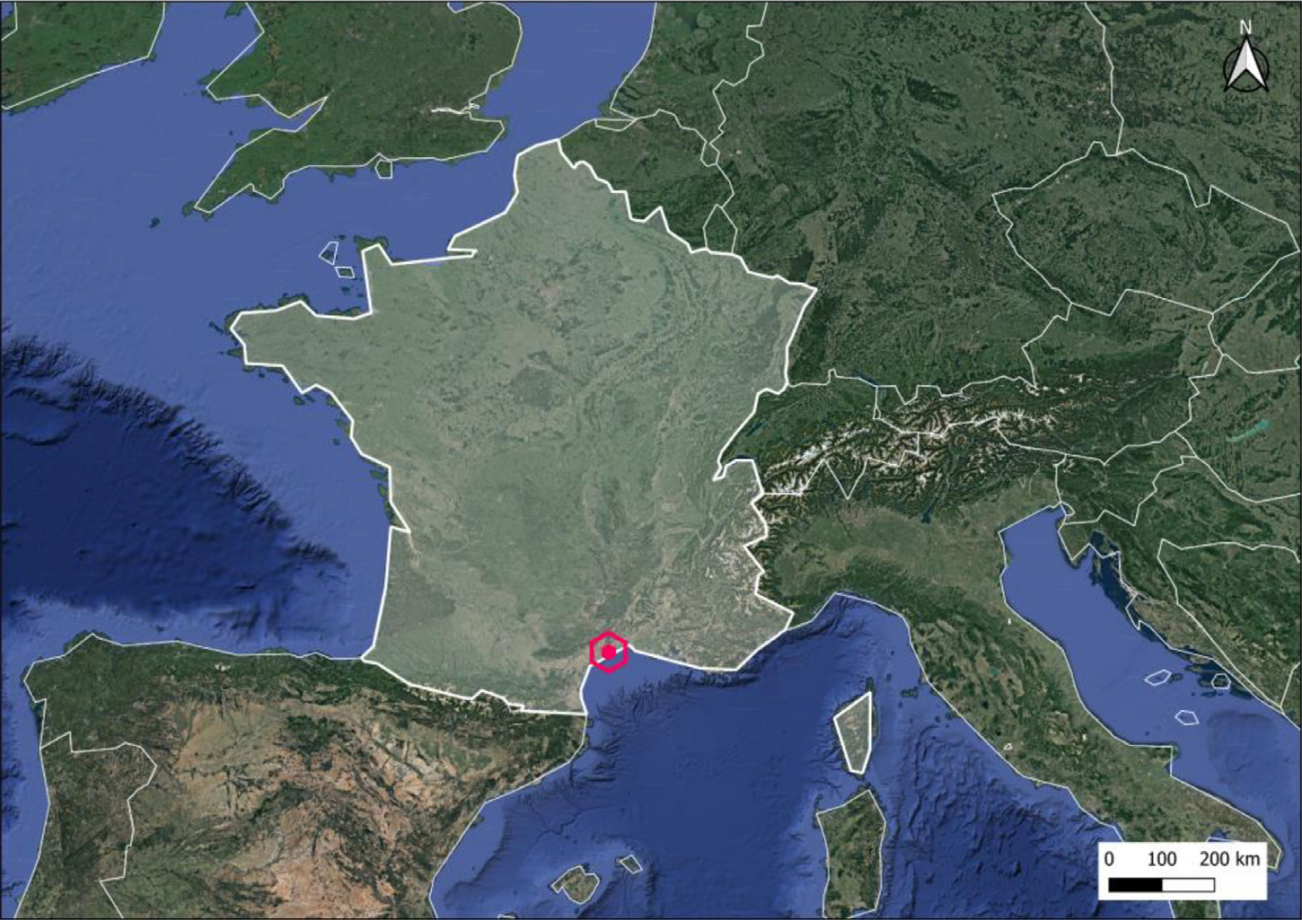
Location of the vineyard on the Mediterranean coast (France). Coordinates: WGS84 - 43.532300° N, 3.864230° E.

**Fig. 2:**
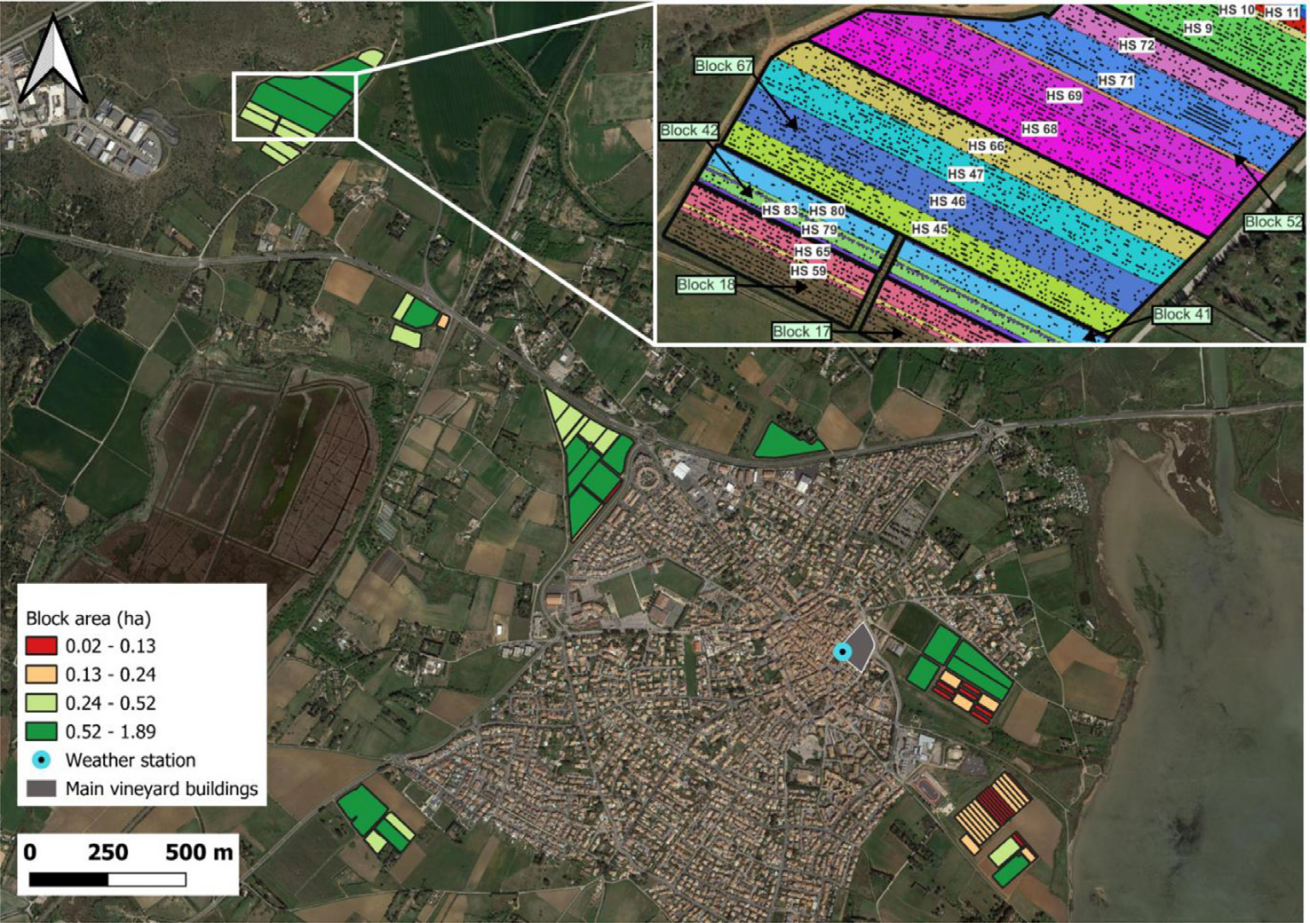
Map of the different blocks of the vineyard, the blocks are colored according to their areas in hectare (ha). Each colour represents an area quartile. The zoom at the top-right illustrates the harvest sectors (each color corresponds to different harvest sectors denoted by “HS XX”) and repartition of unproductive wines within the blocks (each dot refers to an unproductive wine).

The experiment took place in a commercial wine estate located in the south of France (Villeneuve-lès-Maguelone, WGS84 - 43.532300° N, 3.864230° E) in a Mediterranean context (Fig. 1) during the 2022 harvest season (August – September). The area of the vineyard estate is 30 ha organized into 68 different blocks all located within a radius of 3 kilometers around the main vineyard building and the weather station (Fig. 2). Soils over the vineyard are calcisols and cambisols. Soil characteristics are rather homogeneous over the vineyard: the texture is mainly silty clayey sandy, the mean lime content is high (pH = 7.9), the mean organic matter content is moderate (1.78 %) and the soil depth is high for viticulture (> 100 cm with less than 15 % of gravel/stone) which leads to a high water holding capacity (>100 mm).

Block data boundaries and attributes were obtained via the vineyard Farm Management Information System (Fig. 2). Block data boundaries correspond to the regulatory contour used in France to declare the grapes planted areas. The boundary coordinates were measured with a real-time kinematic (RTK) GNSS receiver [1,2]. The main agronomic characteristics of the plots are listed in Table 1. All the plots are grafted and therefore have a rootstock. However, information on the rootstock is not systematically known for each plot. Two main rootstocks are commonly used: Fercal and 110 Richter.

### 3.2 Agronomic data

Yield and harvest quality data were acquired during the 2022 harvest season. Harvesting was performed mechanically with a grape harvesting machine (Pellenc, Pertuis, France) mounted with two hoppers of 600 kg grape capacity each. The harvesting operation was organized in the conventional way; once the harvesting machine was full, the hoppers were emptied into trailers towed to tractors that transferred the grapes to the vineyard cellar or to the wine-growing cooperative cellar. Depending on the distance of the blocks from the cellars, 2 to 3 trailers (with a maximum capacity of 3500 kg of grapes each) were used. At the vineyard cellar, the trailers were systematically weighed and a sample of grape must (30 centiliters) was collected inside the trailer and sent to a commercial laboratory specialized in oenological analysis (Institut Coopératif du Vin, Montpellier, France) to measure harvest quality parameters. Given the filling of the trailer by the harvesting machine, the shakings during transport, and the precautions taken during sampling, this must sample is considered to be representative of the must contained in the whole trailer.At the wine-growing cooperative cellar, the trailers were weighed but no must analysis was performed. The accuracy of the calibrated scales is +/- 50 kg. For each weighing, the time and the date were recorded. Each vineyard vehicle was equipped with a real-time kinematic (RTK) GNSS receiver embedded in a commercial tracking device (TD) manufactured by Samsys company (Lille, France). For this purpose, a static RTK base, part of the Centipède RTK Network [1,2] was installed near the winery, at a maximum distance of 3 km from any of the study blocks. The measured observations (weight of grape and harvest quality parameters) were spatially re-allocate to “harvest sectors” based on the time and the geolocation of the machines involved on the harvesting site. A harvest sector is the area covered by the harvester to fill a harvest trailer before its departure to a cellar. These harvest sectors are zones corresponding to, at most, the harvested area allowed by the capacity of the harvest trailer (see zoom in Fig. 2). Except for very small blocks, they correspond to within block zones. In some rare cases however, an harvest sector may be located on several blocks. The re-allocation of grape parameters was derived from the geolocation recording of all the machines (harvest trailers and grape harvester) during the harvest [6]. This methodology allowed the delimitation of 87 harvest sectors. Among these 87 harvest sectors, 50 corresponds to harvest trailers unloading at the vineyard cellar and 37 to harvest trailers unloading at the wine-growing cooperative cellar. As a result, harvest quality parameters are available only for the 50 harvest sectors that were brought to the vineyard cellar.

Unproductive vines were geolocalized manually over the whole vineyard using a RTK GNSS receiver, of accuracy 1-5 cm (see zoom in Fig. 2).

**Fig. 3:**
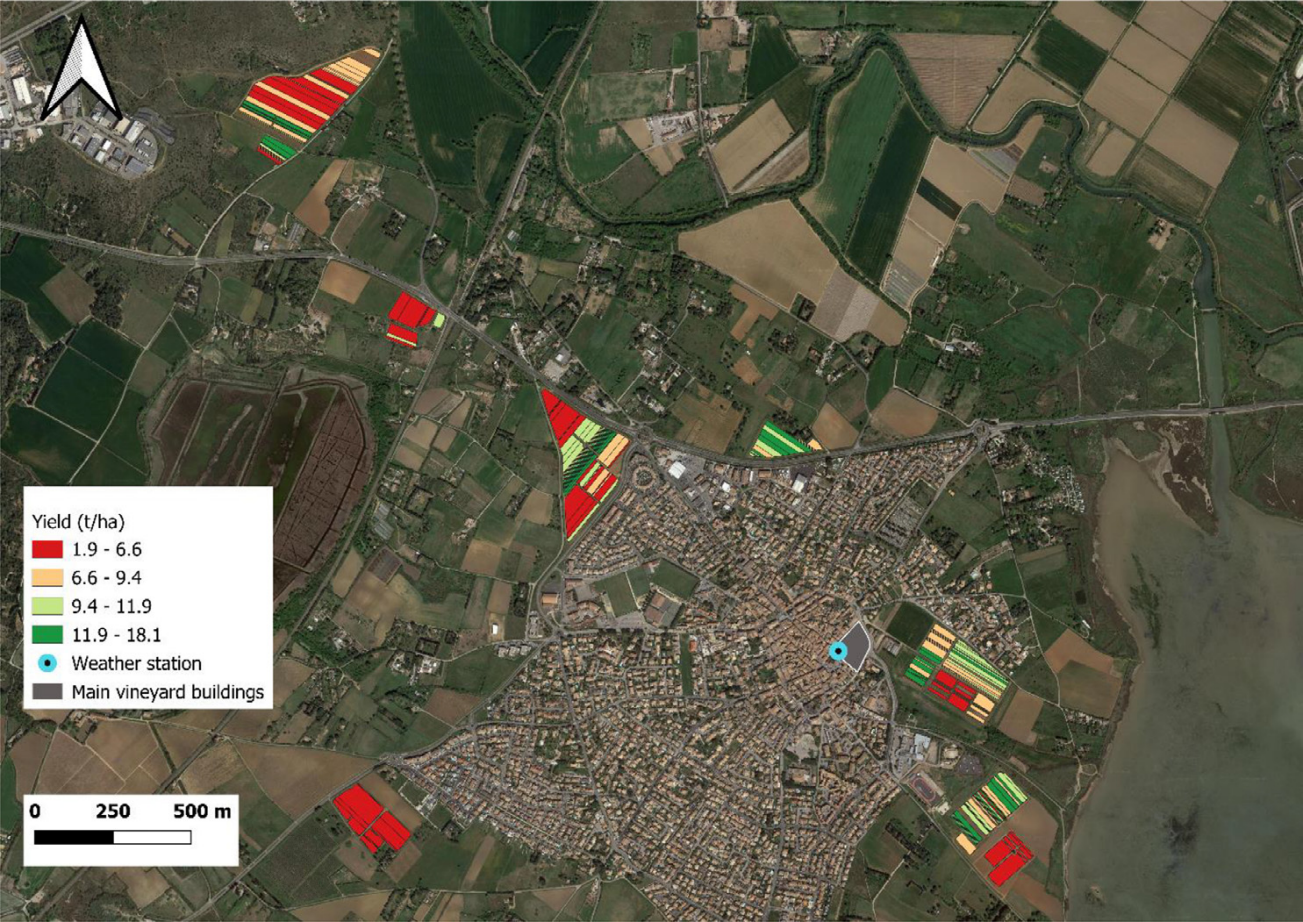
Yield map of harvest sectors (HS) for the whole vineyard estate. Each colour corresponds to a yield quartile calculated at the vineyard estate scale.

**Fig. 4:**
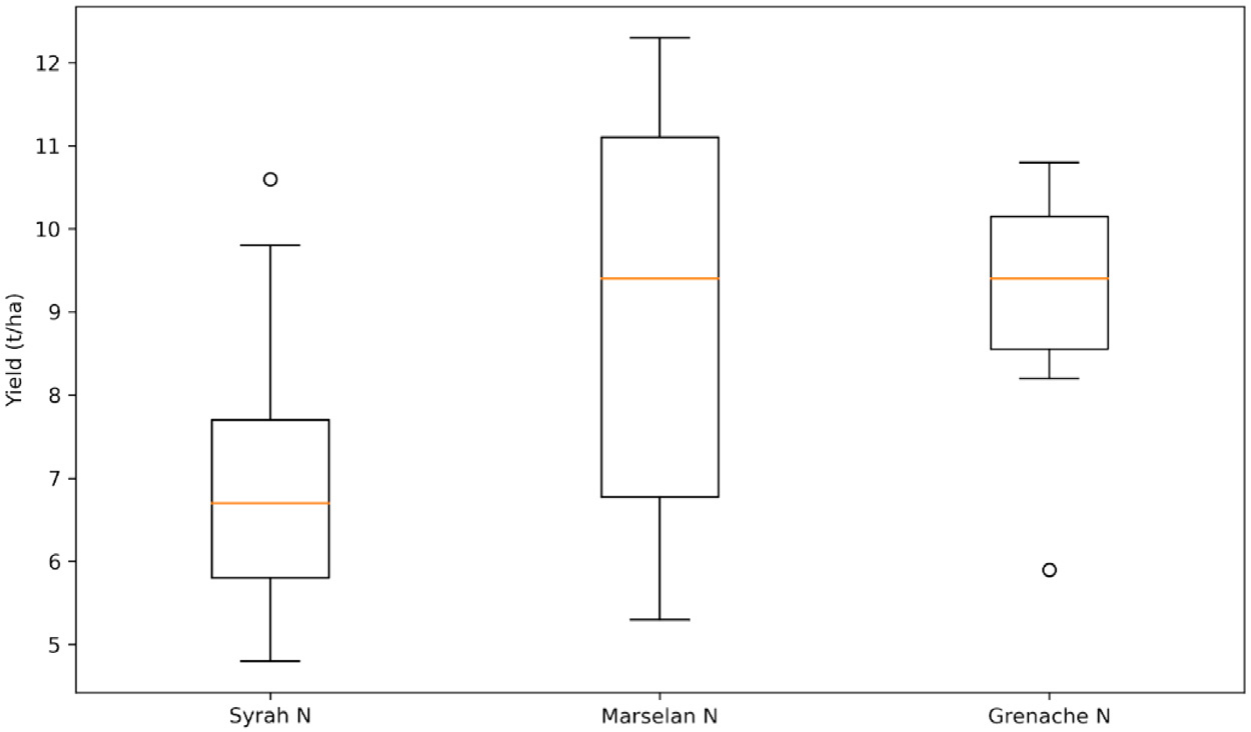
Comparison of the productivity (yield) of 3 grape varieties (Syrah N, Marselan N, Grenache N) over the entire vineyard.

Descriptive parameters of harvest sectors as described in Table 2, were obtained either from measurements (from weighing in the winery and must analysis at the oenological laboratory) or from calculation (yield, unproductive plants, yield per plant). Yield (kg.ha^−1^) of a harvest sector is the mass divided by the harvest sector area. An exemple of yield map is provided on Fig. 3. The percentage of unproductive plants (%) of a harvest sector and denoted by the “UPlants” variable (Table 2), is the number of unproductive plants over the theoretical (derived from the plantation density) number of plants, at the time of the plantation for each considered harvest sector. Yield per plant (kg.plant^−1^) of a harvest sector is denoted “YieldPP” (Table 2). It corresponds to the mass of grapes divided by the number of productive plants of the considered harvest sector. In a harvest sector, the number of productive plants is equal to the original number of plants, at the time of the plantation, minus the number of unproductive plants (in the zoom of Fig. 2, numbers of unproductive plants are illustrated by black dots). A potential use of this agronomical data is presented on Fig. 4. This figure presents the results of the study provided in the “Yield_vs_variety.py” script. On that figure, the productivity (yield) of three grapes varieties over the vineyard is compared.

### 3.3 Weather data

**Fig. 5:**
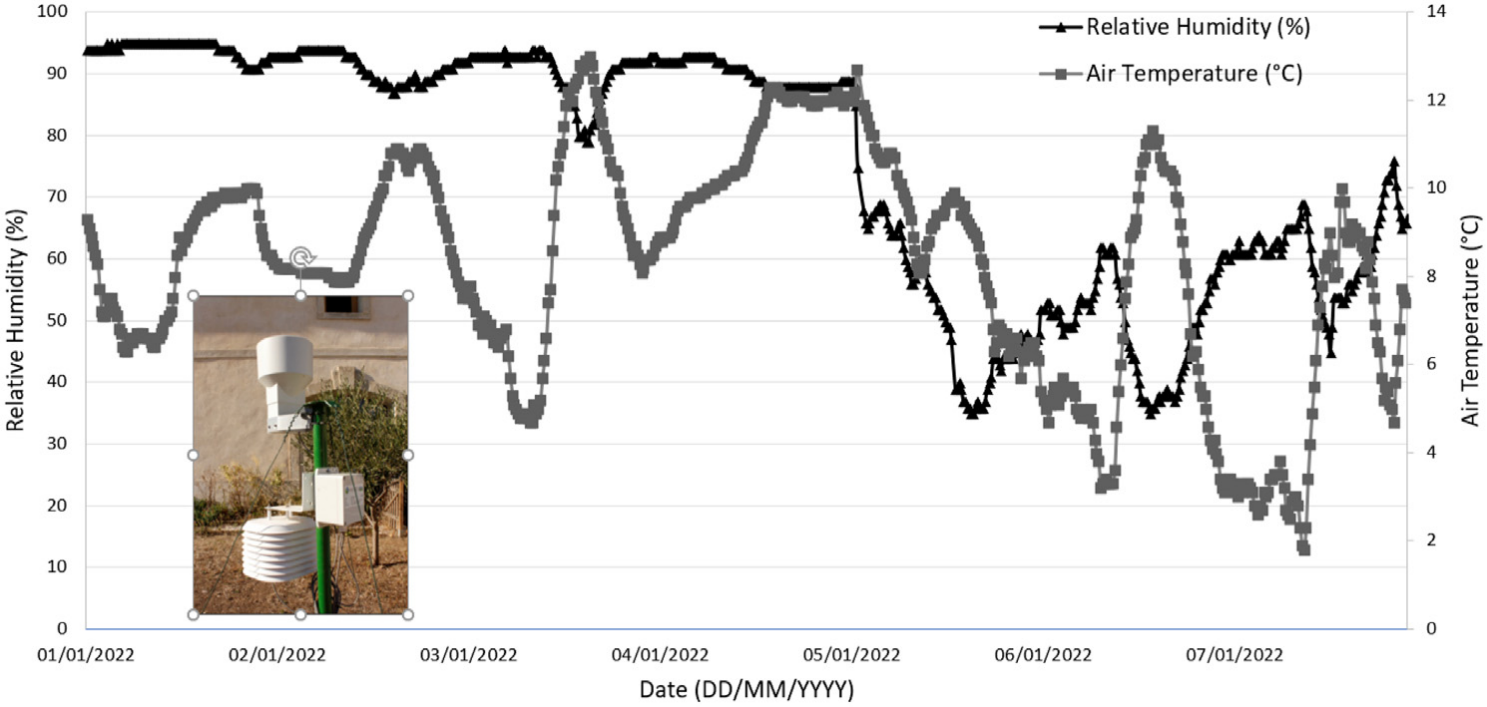
Example of a time series of air temperature and relative humidity (RH) data measured over a week by the weather station (photo inside the graph).

The weather data were provided by a commercial weather station (Agriscope, Montpellier, France) (Fig. 5). It includes several sensors allowing common climatic parameters to be measured; a rain gauge, a thermometer and a hygrometer. The weather station has been installed (43.532096° N, 3.863953° E, WGS 84) in the center of the vineyard estate since 2017. The weather station provides data through the LoRa network based on Low-Power Wide Area Network (LPWAN) technology, to a web server managed by Agriscope company. The data can be consulted online or via an API (application programming interface). From the server, the data can be selected (time interval, data type, etc.) and downloaded in CSV format. The data are provided in CSV format, in the form of a table with all the data from 01/01/2020 to 31/12/2022 with an acquisition frequency of 15 min (Fig. 5) for a total of 97988 measurements. The acquisition campaign has no gaps in the measurements. The weather station is located less than 4 km from any block. The topography of the site is relatively flat (the variation in elevation between the highest and lowest blocks does not exceed 15 m), so the data recorded by the weather station can be considered representative of the climate experienced by the vine plants of every blocks. It should be noted, however, that very local stormy episodes can lead to some variability in rainfall.

## 4 Potential and Limits of the Dataset

The proposed dataset presents some limitations: it concerns a single year of production parameters measurement and it was acquired in a specific region of southern France, with site-specific management practices in non-irrigated conditions. The data observed are also closely related to the local soil conditions. However, such complete data sets are still rare in viticulture, it is of interest for testing, and validating modelling approaches or methods for processing spatial or temporal data in complement with other similar dataset acquired in other regions of the world in different climatic and soil context. The proposed dataset therefore complements any study requiring objective and accurate yield and grape quality data for different grape varieties in viticulture. Its unique feature is to provide these data with a small spatial footprint and therefore a high spatial accuracy. As a complement to datasets acquired in other conditions, it therefore offers potential for gaining a better understanding of the determinants of climate on fruit yield and quality, as in stochastic modelling approaches like those proposed by Laurent et al [4] which aimed at seeking links between climatic time series and observed yield.

The proposed dataset can also be used to extract vegetative indices from remote sensing images (Sentinel 2, Landsat, etc.) from the georeferenced contours of block and harvest sectors. Combining these data with the data measured (yield, sugar, pH, acidity, etc.) is of interest for research aimed at studying the source-sink relationship and the relative abundance of photosynthetically active organs (leaves) with regards to photosynthate demanding organs (mainly fruits and yield) as the main drivers of grape oenological quality [7,8].

Finally, the proposed dataset can be used to extract time series from multispectral remote sensing images (Sentinel 2, Landsat, etc.) based on the provided georeferenced contours. The dataset can therefore be used as a basis for any research work aimed at exploring the potential of image time series as a predictor of yield and quality (with or without accounting for climatic data). The proposed data can be used for methodological research to propose and validate predictive methods such as the functional approaches as proposed by Velez et al [5] or machine learning approaches adapted to small datasets as proposed by Fornieles et al [9].

To our knowledge, there are no data sets of this type published yet in the field of viticulture. With this initiative, the authors hope to promote the publication of similar datasets from other regions of Europe and the world. Indeed, the access to similar data obtained in different climatic and pedological contexts is an important prerequisite for testing and validating the robustness of data processing methods and/or models in order to produce knowledge that is more general.

## Ethics Statements

The authors comply with the ethical guidelines of the journal. Humans, animals, or data from social media are not involved in this research.

## CRediT authorship contribution statement

**Jean-Philippe Gras:** Conceptualization, Methodology, Software, Writing – original draft, Writing – review & editing. **Guilhem Brunel:** Data curation, Writing – original draft. **Arnaud Ducanchez:** Writing – review & editing. **Thomas Crestey:** Conceptualization, Methodology. **Bruno Tisseyre:** Writing – review & editing, Supervision.

## Data Availability

Climatic records and within field data on yield and harvest quality over a whole vineyard estate. (Original data) (Zenodo)
